# A Two Stage Open and Interventional Therapeutic Approach for an Inferior Pancreaticoduodenal Artery Aneurysm With Coeliac Artery Occlusion

**DOI:** 10.1016/j.ejvsvf.2024.06.005

**Published:** 2024-07-03

**Authors:** Polina Shabes, Waseem Garabet, Peter Minko, Joscha Mulorz, Julian-Dario Rembe, Hubert Schelzig, Markus U. Wagenhäuser

**Affiliations:** a Clinic of Vascular and Endovascular Surgery, Medical Faculty and University Hospital Düsseldorf, Germany; b Department of Diagnostic and Interventional Radiology, Medical Faculty and University Hospital Düsseldorf, Germany

**Keywords:** Coeliac trunk occlusion, Coil embolisation, Visceral artery aneurysm, Visceral bypass

## Abstract

**Introduction:**

Visceral artery aneurysms (VAAs) are rare but have a high mortality rate in cases of rupture, especially for pancreaticoduodenal artery aneurysms (PDAAs). A hybrid approach is presented for a challenging case with inferior PDAA (iPDAA) with concomitant coeliac artery (CA) occlusion and a variant arterial supply to the liver.

**Report:**

A 61 year old patient complained of postprandial pain associated with elevated liver enzymes and impaired hepatic synthesis capacity. The left hepatic artery (LHA) originated from an occluded CA, whereas the right hepatic artery (RHA) originated directly from the superior mesenteric artery (SMA) proximal to the iPDAA. Due to the anatomical variant, an endovascular only approach via iPDAA embolisation could have posed a critical risk to the arterial supply of the liver. Therefore, the initial plan was to first secure liver perfusion via endovascular revascularisation of the CA, before conducting a coil embolisation of the iPDAA. However, endovascular CA revascularisation failed due to a complete and fixed occlusion. As an alternative therapeutic approach, open surgical aorto-visceral autologous bypass ensured arterial supply of the liver, which now enabled safe exclusion of the iPDAA via interventional coil embolisation. This two stage hybrid strategy resulted in iPDAA exclusion and was followed by symptom relief and normalised hepatic synthesis capacity.

**Discussion:**

This case demonstrates the continued need for open visceral bypass surgery to ensure organ perfusion, if the latter depends on an aneurysmal artery. In such a situation, visceral bypass surgery can be considered in challenging anatomical scenarios, which demonstrates the relevance of endovascular and open procedures. In conclusion, both procedures can be combined in individualised therapy approaches to maximise patient benefit.

## Highlights

• To demonstrate the ongoing relevance of open surgical skills for treating complex visceral artery aneurysm (VAA).
• To highlight interdisciplinarity to maximize patient benefit.
• To offer a hybrid approach for treatment of iPDAA in complex scenarios.

## Introduction

Visceral artery aneurysms (VAAs) are quite rare,^1^^,^^2^ while pancreaticoduodenal artery aneurysms (PDAAs) only represent a small percentage of VAAs.^1^^,^^3^

Although PDAAs are usually asymptomatic, they require close attention because of their high rupture risk and mortality rate.^4^ Therefore, PDAAs are recommended to be treated regardless of their diameter upon diagnosis.^1^, ^2^, ^3^^,^^5^ However, there is no clear treatment consensus.^6^^,^^8^ Recommended treatment options include coil embolisation, covered stenting, and open surgical reconstruction, if flow is supposed to be preserved.^3^^,^^6^ Furthermore, PDAAs are often associated with coeliac artery (CA) stenosis or occlusion.^3^^,^^7^ This is thought to be related to the wall shear stress hypothesis, which implies increased blood flow in collateral arteries.^1^^,^^7^^,^^8^

In general, endovascular treatment is increasingly recommended in elective and emergency settings due to its lower morbidity and mortality rates.^1^^,^^3^ However, when PDAA is combined with CA occlusion or a variant arterial supply to the liver, an endovascular only approach should be carefully considered. In the following case, the blood flow to the left liver lobe was dependent on the aneurysmal pancreaticoduodenal artery (PDA) due to CA occlusion. Also, the right hepatic artery (RHA) originated from the superior mesenteric artery (SMA) directly proximal to the inferior PDAA (iPDAA).

To exclude the iPDAA while preserving organ perfusion, the patient was treated with a staged hybrid procedure considering the variant arterial liver supply.^4^^,^^6^^,^^9^

## Case report

A 61 year old, male patient with a history of gastritis and appendicectomy presented with occasional epigastric postprandial pain, nausea for four months, and significantly elevated liver enzymes (glutamic oxaloacetic transaminase 424 U/L, glutamic pyruvic transaminase 633 U/L, alkaline phosphatase 106 U/L, bilirubin 1.58 mg/dL). Abdominal ultrasound revealed (asymptomatic) gallstones without signs of cholecystitis. Gastroscopy excluded gastritis. Endoscopic ultrasound and contrast enhanced magnetic resonance imaging revealed an iPDAA of 24 × 32 mm with concomitant CA occlusion. In the referring hospital, the patient was recommended for partial pancreatectomy with iPDAA resection and therefore presented to the outpatient clinic for a second opinion.

An extended gastroenterological diagnostic work up included endoscopic retrograde cholangiopancreatography (ERCP) with fine needle biopsy. The results ruled out malignancy. The patient developed post-ERCP cholecystitis with secondary pancreatitis, which was at first treated conservatively.

**Figure 1:**
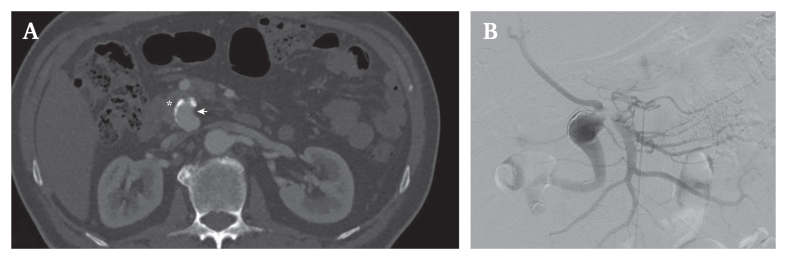
(A) Computed tomography angiography (CTA). The axial pre-surgical CTA view showed an inferior pancreaticoduodenal artery (iPDAA) (arrow) in close proximity to the pancreatic head (∗). (B) Digital subtraction angiography (DSA). The DSA confirmed a complete chronic occlusion of the coeliac artery (CA), which appeared independent of respiratory movements (not shown). The endovascular revascularisation attempt failed. The DSA further confirmed the iPDAA with a variant arterial blood supply of the liver. The right hepatic artery (RHA) arises directly proximal to the iPDAA from the superior mesenteric artery (SMA).

**Figure 2:**
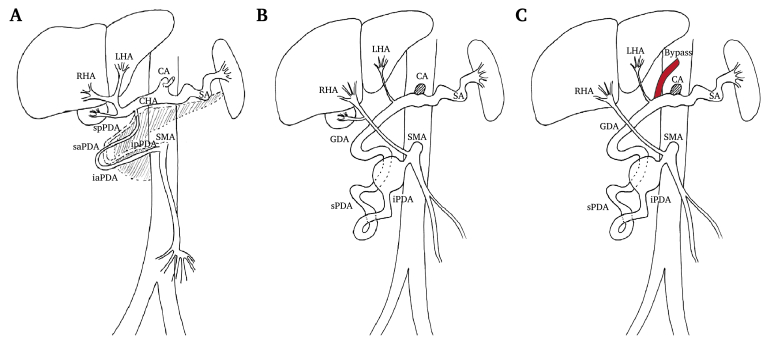
Schematic illustration of the anatomical conditions. (A) Schematic illustration of the normal anatomical conditions. The spatial position of the pancreas is shown by a hatched area. (B) Pre-surgical anatomical situation. The coeliac artery (CA) was chronically occluded. The right hepatic artery (RHA) arises directly proximal to the inferior pancreaticoduodenal artery aneurysm (iPDAA) from the superior mesenteric artery (SMA). The left hepatic artery (LHA) arises from the CA and is retrogradely perfused from the SMA over the inferior pancreaticoduodenal artery (iPDA) with its proximal iPDAA. (C) Post-surgical anatomical situation. An autologous reversed great saphenous vein aortovisceral bypass was placed to provide antegrade perfusion to the occluded CA. The bypass secured arterial liver perfusion for the time delayed interventional exclusion of the iPDAA by coil embolisation in case of coil associated complications. Red: aortovisceral bypass. GDA = gastroduodenal artery; sPDA = superior pancreaticoduodenal artery; iaPDA = inferior anterior pancreaticoduodenal artery; ipPDA = inferior posterior pancreaticoduodenal artery; saPDA = superior anterior pancreaticoduodenal artery; spPDA = superior posterior pancreaticoduodenal artery; SA = splenic artery.

After a multidisciplinary consultation, an endovascular only approach was chosen by first opening the CA and embolising the iPDAA afterwards to secure left liver lobe perfusion. Nevertheless, in line with the computed tomography angiography (CTA) (Fig. 1A), digital substraction angiography revealed a complete chronic CA occlusion (unaffected by respiratory movements^5^), a large-caliber PDA with an proximal iPDAA (Fig. 1B), and a variant arterial blood supply to the liver (Fig. 1B, Fig. 2A and B). The iPDAA was located in close proximity to the origin of the RHA, which originated directly from the SMA (Fig. 1B). The left hepatic artery (LHA) contrasted retrogradely via the large-calibre PDA. After multiple attempts, re-opening the CA was considered impossible.

Considering the risk of liver ischaemia or other pancreatic related complications,^10^ a two stage hybrid approach with open surgical CA revascularisation was recommended to secure an antegrade arterial supply to the liver first. A bypass was created from the aorta to the LHA using a reversed great saphenous vein graft via a median laparatomy and a transbursal approach (Fig. 2C). In addition, a cholecystectomy was performed, since the gallbladder was found oedematous during surgery due to the post-ERCP cholecystitis two months before. After surgery, the patient was admitted to the intensive care unit (ICU) and anticoagulation was started to secure graft patency.

On the first post-operative day, re-do surgery with direct suturing was necessary due to bleeding from the posterior side of the autologous vein bypass. Two days after re-do surgery, the patient was transferred to the regular ward. The further post-operative course was uncomplicated.

**Figure 3:**
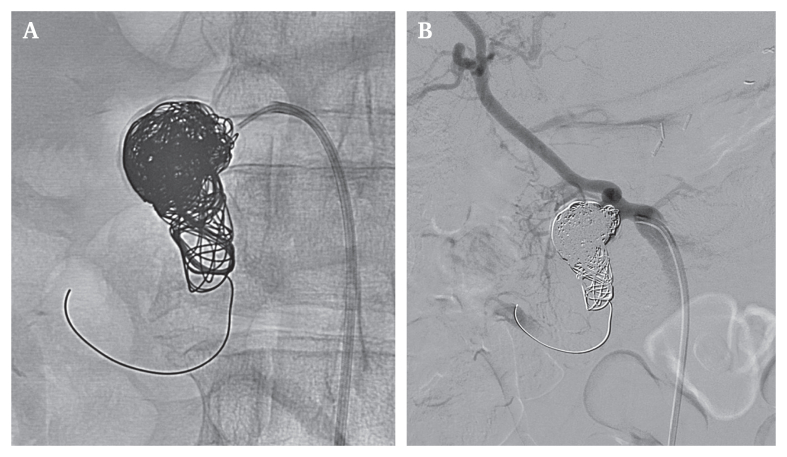
Coil embolisation of the of the inferior pancreaticoduodenal artery aneurysm (iPDAA). (A) Correct position of the coils in the iPDAA during a transfemoral approach for interventional coil embolisation. (B) Digital subtraction angiography (DSA) illustrates the perfusion of the right hepatic artery (RHA) which arises directly proximal to the now excluded iPDAA. DSA also confirmed no thromboembolic event during the procedure. The (inferior) pancreaticoduodenal artery ((i)PDA) was occluded intentionally (the guide wire remained in the vessel).

Three weeks later, the iPDAA was excluded by transfemoral coil embolisation using 13 Cosmos coils (MicroVention-Terumo, Aliso Viejo, CA, USA), two helical coils, 10 IDC-Interlock coils (Boston Scientific, Marlborough, MA, USA), one framing coil, and two HydroCoils (Terumo Medical Corp., Somerset, NJ, USA). The final two coils of 8 cm length (2 ×5 mm and 1 × 4 mm) were placed into the iPDAA neck. Altogether 28 coils were used during the interventional procedure, being carefully placed to avoid aneurysm rupture or accidental occlusion of origin of the nearby RHA, while ensuring complete aneurysm occlusion. The procedure progressed without complications and confirmed a patent aortovisceral bypass and a completely excluded iPDAA. Also, adequate liver perfusion via the open LHA and RHA was confirmed (Fig. 3). After a complication free post-interventional course, the patient was discharged a few days later.

After follow-up of more than a year the patient reported subjective wellbeing and significant weight gain (>4 kg). Blood tests showed complete normalisation of liver enzymes. The patient is currently on antiplatelet therapy (aspirin) and oral anticoagulation, given the significant impact of liver failure in cases of bypass occlusion. A follow-up CTA scan confirmed the patency of the aortovisceral bypass and successful iPDAA exclusion. The patient provided written informed consent for publication of this case.

## Discussion

This case involved a CA occlusion with an iPDAA directly located on the path providing arterial perfusion to the left liver lobe. An isolated endovascular only exclusion of the iPDAA might have resulted in left liver lobe ischaemia, which was too great a risk for the already impaired liver function.^3^^,^^4^ When considering endovascular iPDAA treatment, the tortuous iPDA and the close proximity of the RHA origin to the aneurysm neck prevented an adequate landing zone for stent placement and further increased the risk of liver ischaemia in the case of coil displacement.^6^ Direct open surgical iPDAA resection could have increased the anastomosis related complication risk significantly in this elective case, considering the close proximity to the pancreatic head and pancreaticoduodenal junction.^10^ For these reasons, a hybrid approach was favoured. An open surgical approach was chosen to create an autologous venous bypass from the aorta to LHA aiming to first secure liver perfusion before endovascular coil embolisation of the iPDAA.^3^^,^^4^^,^^8^

Currently, there are several strategies for the treatment of CA stenosis and or occlusion with only limited evidence regarding the most beneficial therapeutic approach. In the currently available literature, most authors prefer an endovascular first approach, while open surgical revascularisation has been mentioned in the context of inadequate end organ perfusion.^4^^,^^8^ A first diagnostic step is CTA to define the grade of calcification and the length of the pathology.^3^^,^^5^ In the present case, a fixed CA occlusion was observed, which could not be revascularised, so it was necessary to place an aortovisceral bypass.

Currently, mainly endovascular procedures are used to treat VAAs^1^^,^^3^ given their high procedural success rate. Nevertheless, the risk of thromboembolic events, embolisation of a non-target vessel, and coil migration is not negligible.^6^ In the present case, such events could have completely blocked arterial liver perfusion, and therefore a two stage hybrid approach with open surgical aortovisceral bypass was chosen to revascularise the CA first, aiming to ensure liver perfusion in the case of possible thromboembolic events during iPDAA embolisation.^4^ The interval between the open surgical and endovascular procedure confirmed the patency of the aortohepatic bypass with no evidence of post-operative complications such as bypass occlusion or kinking.

Such a cautious approach optimised patient safety and proved successful in terms of complete symptomatic relief. Alternatively, iPDAA resection could have been performed during open surgical bypass in a single stage therapeutic approach, a commonly used option. Disadvantageously, in this case, it would have increased the complication risk and might even have required partial pancreatectomy.^10^ In this case it may be discussed whether an isolated CA occlusion without stenosis of the mesenteric arteries is sufficient to cause symptoms of chronic mesenteric ischaemia^9^ and whether the performed cholecystectomy might have contributed to the improvement of clinical symptoms and blood values. Yet, according to earlier case reports, CA revascularisation and (i)PDAA exclusion (without cholecystectomy) lead to symptom relief.^4^

In the present case, the patient received both antiplatelet therapy (aspirin) and oral anticoagulation (phenprocoumon) given the initially narrow diameter of the great saphenous vein in order to prevent aortohepatic bypass occlusion with severe consequences.

### Conclusions

In conclusion, a challenging and rare case of an iPDAA at a complex location, complicated by a variant arterial supply to the liver, is presented. This demonstrates the continued need for an individual therapeutic approach, involving open surgery only or, like in this case, visceral bypass surgery in combination with interventional solutions such as endovascular coil embolisation. The present case is an example of profitable interdisciplinary cooperation within an individualised treatment approach that maximised patient benefit.

## Conflict of interest

None.

## References

[1] Bonardelli S., Spampinato B., Ravanelli M., Cuomo R., Zanotti C., Paro B. (2020). The role of emergency presentation and revascularization in aneurysms of the peripancreatic arteries secondary to celiac trunk or superior mesenteric artery occlusion. J Vasc Surg.

[2] Illuminati G., Hostalrich A., Pasqua R., Nardi P., Chaufour X., Ricco J.B. (2021). Outcomes after open and endovascular repair of non-ruptured true pancreaticoduodenal and gastroduodenal artery aneurysms associated with coeliac artery compression: a multicentre retrospective study. Eur J Vasc Endovasc Surg.

[3] Chaer R.A., Abularrage C.J., Coleman D.M., Eslami M.H., Kashyap V.S., Rockman C. (2020). The Society for Vascular Surgery clinical practice guidelines on the management of visceral aneurysms. J Vasc Surg.

[4] Deser S.B., Demirag M.K. (2017). Surgical treatment of inferior pancreaticoduodenal artery aneurysm with common hepatic artery revascularization. Ann Vasc Surg.

[5] Björck M., Koelemay M., Acosta S., Bastos Goncalves F., Kölbel T., Kolkman J.J. (2017). Editor's Choice – management of the diseases of mesenteric arteries and veins: clinical practice guidelines of the European Society of Vascular Surgery (ESVS). Eur J Vasc Endovasc Surg.

[6] Cordova A.C., Sumpio B.E. (2013). Visceral artery aneurysms and pseudoaneurysms—should they all be managed by endovascular techniques?. Ann Vasc Dis.

[7] Miyahara K., Hoshina K., Nitta J., Kimura M., Yamamoto S., Ohshima M. (2019). Hemodynamic simulation of pancreaticoduodenal artery aneurysm formation using an electronic circuit model and a case series analysis. Ann Vasc Dis.

[8] Aryal B., Komokata T., Ueno T., Yamamoto B., Senokuchi T., Yasuda H. (2017). A 2-stage surgical and endovascular treatment of rare multiple aneurysms of pancreatic arteries. Ann Vasc Surg.

[9] Huber T.S., Björck M., Chandra A., Clouse W.D., Dalsing M.C., Oderich G.S. (2021). Chronic mesenteric ischemia: clinical practice guidelines from the society for vascular surgery. J Vasc Surg.

[10] Malleo G., Vollmer Jr CM. (2016). Postpancreatectomy complications and management. Surg Clin North Am.

