# Supplementary material for: A Two Stage Open and Interventional Therapeutic Approach for an Inferior Pancreaticoduodenal Artery Aneurysm With Coeliac Artery Occlusion
Source: EJVES Vasc Forum. 2024 Jul 3;62:25–9. doi: 10.1016/j.ejvsvf.2024.06.005 (PMC11419828; doi:10.1016/j.ejvsvf.2024.06.005)
Supplement: Multimedia component 1. [file mmc1.pdf]

Supplementary Table

Literature overview. The table presents reports of treatments of pancreaticoduodenal artery aneurysm (PDA) with complicating celiac artery (CA) stenosis or occlusion (without any claim to completeness). ER = endovascular repair; OR = open repair; PDA = pancreaticoduodenal artery; PDAA = pancreaticoduodenal artery aneurysm; GDA = gastroduodenal artery; CA = celiac artery.

Antoniak et al., 2018	Retrospective case study of aneurysms involving every hepatic perfusion pathway – only-surgical vs hybrid approach.
Aryal et al., 2017	Case report with a combination of a CA stenosis and aneurysms of the dorsal pancreatic artery and anterior inferior pancreaticoduodenal artery.
Boll et al., 2017	Retrospective study on PDA and GDA treatment ER and OR.
Bonardelly et al., 2020	Retrospectively (10 years period) analysed cases of pancreaticoduodenal artery aneurysms after steno-occlusion of the celiac trunk and superior mesenteric artery emphasizing the need for an individual open surgical and/or endovascular approach.
Brocker et al., 2012	Case report and treatment discussion on PDAA rupture and celiac axis revascularization.
Deser et al., 2017	Case report with a combination of celiac axis occlusion and PDAA – solution with hybrid approach.
Ducasse et al., 2004	Case report on PDA and CA compression by median arcuate ligament division.
Flood and Nicholson, 2013	Retrospective case study and review on different treatment options of PDA aneurysm and CA stenosis or occlusion – ER and OR.
Franke et al., 2021	Case report on CA stenosis and PDAA.
Hughes et al., 2016	Case report on aorto-hepatic bypassing for CA occlusion and PDA aneurysm resection.
Ikeda et al., 2013	Retrospective study on ER via PDA aneurysm coiling and or CA stenting for revascularization.
Illuminati et al., 2021	Retrospective study discussing PDAA treatment: Endovascular vs. Open Repair.
Kalva et al., 2007	Review article on iPDA aneurysms and CA stenosis or occlusion.
Kamarajah et al., 2019	Case report on PDA aneurysm coiling and CA stenosis without revascularization.
Kubota et al., 2022	Case series with surgical celiac revascularization and PDAA resection or coiling.
Kwag et al., 2020	Case series on endovascular PDA aneurysm coiling
Lim et al., 2023	Case report on ruptured PDAA with CA stenosis, emergency coiling.
Miyahara et al., 2019	Publication describing a theoretical model for aneurysm development after celiac trunk occlusion.
Sutton and Lawton 1973	Publication first describing a connection between CA stenosis or occlusion and aneurysm development within the collateral supply arteries.
Takeuchi et al., 2017	Case report series on CA stenosis with different therapeutic options: ER and OR and PDA aneurysm coiling.
Uher et al., 1994	Case report series demonstrating several therapeutic options.
Zhang et al, 2019	Case report on the combination of CA stenosis and ruptured PDA aneurysm – Solution: endovascular aneurysm embolization and celiac trunk stenting.

References for the Supplementary Table:

1) Antoniak R, Grabowska-Derlatka L, Maciag R, Ostrowski T, Nawrot I, Galazka Z, Nazarewski S, Rowinski O. Treatment Algorithm of Peripancreatic Arteries Aneurysm Coexisting with Coeliac Artery Lesion: Single Institution Experience. Biomed Res Int. 2018;2018:5745271.
2) Aryal B, Komokata T, Ueno T, Yamamoto B, Senokuchi T, Yasuda H, Kaieda M, Imoto Y. A 2-Stage Surgical and Endovascular Treatment of Rare Multiple Aneurysms of Pancreatic Arteries. Ann Vasc Surg. 2017;40:295.e9-295.e13.
3) Boll JM, Sharp KW, Garrard CL, Naslund TC, Curci JA, Valentine RJ. Does Management of True Aneurysms of Peripancreatic Arteries Require Repair of Associated Celiac Artery Stenosis? J Am Coll Surg. 2017;224:199-203.
4) Bonardelli S, Spampinato B, Ravanelli M, Cuomo R, Zanotti C, Paro B et al. The role of emergency presentation and revascularization in aneurysms of the peripancreatic arteries secondary to celiac trunk or superior mesenteric artery occlusion. J Vasc Surg. 2020;72:46S-55S.
5) Brocker, JA, Maher JL, Smith RW. True pancreaticoduodenal aneurysms with celiac stenosis or occlusion. Am J Surg. 2012;204:762-8.
6) Deser SB, Demirag MK. Surgical Treatment of Inferior Pancreaticoduodenal Artery Aneurysm with Common Hepatic Artery Revascularization. Ann Vasc Surg. 2017;43:313.e9-313.e11.
7) Ducasse E, Roy F, Chevalier J, Massouille D, Smith M, Speziale S, Fiorani P, Puppinck P. Aneurysm of the pancreaticoduodenal arteries with a celiac trunk lesion: current management. J Vasc Surg. 2004 Apr;39(4):906-11.
8) Flood K, Nicholson AA. Inferior pancreaticoduodenal artery aneurysms associated with occlusive lesions of the celiac axis: diagnosis, treatment options, outcomes, and review of the literature. Cardiovasc Intervent Radiol. 2013;36:578-87.
9) Franke M, Mückner K. Pankreatikoduodenale Aneurysmen in Kombination mit einer Stenose des Truncus coeliacus („Sutton-Kadir-Syndrom“). Rofo. 2021;193:1218-1219.
10) Hughes T, Chatzizacharias NA, Richards J, Harper S. Aorto-hepatic bypass graft for repair of an inferior pancreatico-duodenal artery aneurysm associated with coeliac axis occlusion: A case report. Int J Surg Case Rep. 2016;28:131-134.
11) Ikeda O, Nakasone Y, Yokoyama K, Inoue S, Tamura Y, Yamashita Y. Simultaneous coil embolization and angioplasty using a self-expanding nitinol stent to treat pancreaticoduodenal artery aneurysms associated with celiac artery stenosis. Acta Radiol. 2013;54:949-53.
12) Illuminati G, Hostalrich A, Pasqua R, Nardi P, Chaufour X, Ricco JB. Outcomes After Open and Endovascular Repair of Non-Ruptured True Pancreaticoduodenal and Gastroduodenal Artery Aneurysms Associated with Coeliac Artery Compression: A Multicentre Retrospective Study. Eur J Vasc Endovasc Surg. 2021;61:945-953.
13) Kalva SP, Athanasoulis CA, Greenfield AJ, Fan CM, Curvelo M, Waltman AC, Wicky S. Inferior pancreaticoduodenal artery aneurysms in association with celiac axis stenosis or occlusion. Eur J Vasc Endovasc Surg. 2007;33:670-5.
14) Kamarajah SK, Kharkhanis S, Duddy M, Isaac J, Sutcliffe RP, Mehrzad H, Dasari BVM. Management of pancreaticoduodenal artery aneurysm associated with coeliac artery stenosis. Ann R Coll Surg Engl. 2019;101:e105-e107.
15) Kubota K, Shimizu A, Notake T, Wada Y, Soejima Y. Treatment strategies for unruptured pancreaticoduodenal artery aneurysms associated with celiac artery occlusion. Ann Gastroenterol Surg. 2022;7:182-189.
16) Kwag M, Jung HS, Heo YJ, Baek JW, Shin GW. Embolization of Inferior Pancreaticoduodenal Artery Aneurysm with Celiac Stenosis or Occlusion: A Report of Three Cases and a Review of Literature. Taehan Yongsang Uihakhoe Chi. 2020;81:945-952.
17) Lim J, De Robles MS, Putnis S. Pancreaticoduodenal artery aneurysm associated with coeliac stenosis: a potential bomb that carries no warning. ANZ J Surg. 2023;93:1367-1368.
18) Miyahara K, Hoshina K, Nitta J, Kimura M, Yamamoto S, Ohshima M. Hemodynamic Simulation of Pancreaticoduodenal Artery Aneurysm Formation Using an Electronic Circuit Model and a Case Series Analysis. Ann Vasc Dis. 2019;12:176-181.
19) Sutton D, Lawton G. Coeliac stenosis or occlusion with aneurysm of the collateral supply. Clin Radiol. 1973;24:49-53.
20) Takeuchi Y, Morikage N, Samura M, Harada T, Yamashita O, Suehiro K, Okada M, Hamano K. Treatment Options for Celiac Stenosis and Pancreaticoduodenal Artery Aneurysms. Ann Vasc Surg. 2017:41:281.e21-281.e23.
21) Uher P, Nyman U, Ivancev K, Lindh M. Aneurysms of the pancreaticoduodenal artery associated with occlusion of the celiac artery. Abdom Imaging. 1995;20:470-3.

22) Zhang XZ, Zhang W, Zhou W, Zhou W. Endovascular Treatment of Ruptured Pancreaticoduodenal Artery Aneurysm with Celiac Axis Stenosis. Ann Vasc Surg. 2019;57:273.e1-273.e5.
