# Supplementary material for: A Two Stage Open and Interventional Therapeutic Approach for an Inferior Pancreaticoduodenal Artery Aneurysm With Coeliac Artery Occlusion
Source: EJVES Vasc Forum. 2024 Jul 3;62:25–9. doi: 10.1016/j.ejvsvf.2024.06.005 (PMC11419828; doi:10.1016/j.ejvsvf.2024.06.005)
Supplement: Multimedia component 2. [file mmc2.docx]

**Supplementary Table**

Literature overview. The table presents reports of treatments of pancreaticoduodenal artery aneurysm (PDA) with complicating celiac artery (CA) stenosis or occlusion (without any claim to completeness). ER = endovascular repair; OR = open repair; PDA = pancreaticoduodenal artery; PDAA = pancreaticoduodenal artery aneurysm; GDA = gastroduodenal artery; CA = celiac artery.

| Antoniak et al., 2018 | Retrospective case study of aneurysms involving every hepatic perfusion pathway – only-surgical vs hybrid approach. |
| --- | --- |
| Aryal et al., 2017 | Case report with a combination of a CA stenosis and aneurysms of the dorsal pancreatic artery and anterior inferior pancreaticoduodenal artery. |
| Boll et al., 2017 | Retrospective study on PDA and GDA treatment ER and OR. |
| Bonardelly et al., 2020 | Retrospectively (10 years period) analysed cases of pancreaticoduodenal artery aneurysms after steno-occlusion of the celiac trunk and superior mesenteric artery emphasizing the need for an individual open surgical and/or endovascular approach. |
| Brocker et al., 2012 | Case report and treatment discussion on PDAA rupture and celiac axis revascularization. |
| Deser et al., 2017 | Case report with a combination of celiac axis occlusion and PDAA – solution with hybrid approach. |
| Ducasse et al., 2004 | Case report on PDA and CA compression by median arcuate ligament division. |
| Flood and Nicholson, 2013 | Retrospective case study and review on different treatment options of PDA aneurysm and CA stenosis or occlusion – ER and OR. |
| Franke et al., 2021 | Case report on CA stenosis and PDAA. |
| Hughes et al., 2016 | Case report on aorto-hepatic bypassing for CA occlusion and PDA aneurysm resection. |
| Ikeda et al., 2013 | Retrospective study on ER via PDA aneurysm coiling and or CA stenting for revascularization. |
| Illuminati et al., 2021 | Retrospective study discussing PDAA treatment: Endovascular vs. Open Repair. |
| Kalva et al., 2007 | Review article on iPDA aneurysms and CA stenosis or occlusion. |
| Kamarajah et al., 2019 | Case report on PDA aneurysm coiling and CA stenosis without revascularization. |
| Kubota et al., 2022 | Case series with surgical celiac revascularization and PDAA resection or coiling. |
| Kwag et al., 2020 | Case series on endovascular PDA aneurysm coiling |
| Lim et al., 2023 | Case report on ruptured PDAA with CA stenosis, emergency coiling. |
| Miyahara et al., 2019 | Publication describing a theoretical model for aneurysm development after celiac trunk occlusion. |
| Sutton and Lawton 1973 | Publication first describing a connection between CA stenosis or occlusion and aneurysm development within the collateral supply arteries. |
| Takeuchi et al., 2017 | Case report series on CA stenosis with different therapeutic options: ER and OR and PDA aneurysm coiling. |
| Uher et al., 1994 | Case report series demonstrating several therapeutic options. |
| Zhang et al, 2019 | Case report on the combination of CA stenosis and ruptured PDA aneurysm – Solution: endovascular aneurysm embolization and celiac trunk stenting. |

**References for the Supplementary Table:**

1. Antoniak R, Grabowska-Derlatka L, Maciag R, Ostrowski T, Nawrot I, Galazka Z, Nazarewski S, Rowinski O. Treatment Algorithm of Peripancreatic Arteries Aneurysm Coexisting with Coeliac Artery Lesion: Single Institution Experience. Biomed Res Int. 2018;2018:5745271.
2. Aryal B, Komokata T, Ueno T, Yamamoto B, Senokuchi T, Yasuda H, Kaieda M, Imoto Y. A 2-Stage Surgical and Endovascular Treatment of Rare Multiple Aneurysms of Pancreatic Arteries. Ann Vasc Surg. 2017;40:295.e9-295.e13.
3. Boll JM, Sharp KW, Garrard CL, Naslund TC, Curci JA, Valentine RJ. Does Management of True Aneurysms of Peripancreatic Arteries Require Repair of Associated Celiac Artery Stenosis? J Am Coll Surg. 2017;224:199-203.
4. Bonardelli S, Spampinato B, Ravanelli M, Cuomo R, Zanotti C, Paro B et al. The role of emergency presentation and revascularization in aneurysms of the peripancreatic arteries secondary to celiac trunk or superior mesenteric artery occlusion. J Vasc Surg. 2020;72:46S-55S.
5. Brocker, JA, Maher JL, Smith RW. True pancreaticoduodenal aneurysms with celiac stenosis or occlusion. Am J Surg. 2012;204:762-8.
6. Deser SB, Demirag MK. Surgical Treatment of Inferior Pancreaticoduodenal Artery Aneurysm with Common Hepatic Artery Revascularization. Ann Vasc Surg. 2017;43:313.e9-313.e11.
7. Ducasse E, Roy F, Chevalier J, [Massouille](https://pubmed.ncbi.nlm.nih.gov/?sort=date&term=Massouille+D&cauthor_id=15071464) D, Smith M, Speziale S, Fiorani P, [Puppinck](https://pubmed.ncbi.nlm.nih.gov/?sort=date&term=Puppinck+P&cauthor_id=15071464) P. Aneurysm of the pancreaticoduodenal arteries with a celiac trunk lesion: current management. J Vasc Surg. 2004 Apr;39(4):906-11.
8. Flood K, Nicholson AA. Inferior pancreaticoduodenal artery aneurysms associated with occlusive lesions of the celiac axis: diagnosis, treatment options, outcomes, and review of the literature. Cardiovasc Intervent Radiol. 2013;36:578-87.
9. Franke M, Mückner K. Pankreatikoduodenale Aneurysmen in Kombination mit einer Stenose des Truncus coeliacus („Sutton-Kadir-Syndrom“). Rofo. 2021;193:1218-1219.
10. Hughes T, [Chatzizacharias](https://pubmed.ncbi.nlm.nih.gov/?sort=date&term=Chatzizacharias+NA&cauthor_id=27701003) NA, Richards J, Harper S. Aorto-hepatic bypass graft for repair of an inferior pancreatico-duodenal artery aneurysm associated with coeliac axis occlusion: A case report. Int J Surg Case Rep. 2016;28:131-134.
11. Ikeda O, Nakasone Y, Yokoyama K, Inoue S, Tamura Y, Yamashita Y. Simultaneous coil embolization and angioplasty using a self-expanding nitinol stent to treat pancreaticoduodenal artery aneurysms associated with celiac artery stenosis. Acta Radiol. 2013;54:949-53.
12. Illuminati G, [Hostalrich](https://pubmed.ncbi.nlm.nih.gov/?sort=date&size=50&term=Hostalrich+A&cauthor_id=33762153) A, Pasqua R, Nardi P, [Chaufour](https://pubmed.ncbi.nlm.nih.gov/?sort=date&size=50&term=Chaufour+X&cauthor_id=33762153) X,  Ricco JB. Outcomes After Open and Endovascular Repair of Non-Ruptured True Pancreaticoduodenal and Gastroduodenal Artery Aneurysms Associated with Coeliac Artery Compression: A Multicentre Retrospective Study. Eur J Vasc Endovasc Surg. 2021;61:945-953.
13. Kalva SP, [Athanasoulis](https://pubmed.ncbi.nlm.nih.gov/?sort=date&term=Athanasoulis+CA&cauthor_id=17276102) CA, Greenfield AJ, Fan CM, Curvelo M, Waltman AC, Wicky S. Inferior pancreaticoduodenal artery aneurysms in association with celiac axis stenosis or occlusion. Eur J Vasc Endovasc Surg. 2007;33:670-5.
14. Kamarajah SK, [Kharkhanis](https://pubmed.ncbi.nlm.nih.gov/?sort=date&term=Kharkhanis+S&cauthor_id=30855165) S, [Duddy](https://pubmed.ncbi.nlm.nih.gov/?sort=date&term=Duddy+M&cauthor_id=30855165) M, Isaac J, [Sutcliffe](https://pubmed.ncbi.nlm.nih.gov/?sort=date&term=Sutcliffe+RP&cauthor_id=30855165) RP, [Mehrzad](https://pubmed.ncbi.nlm.nih.gov/?sort=date&term=Mehrzad+H&cauthor_id=30855165) H, [Dasari](https://pubmed.ncbi.nlm.nih.gov/?sort=date&term=Dasari+B&cauthor_id=30855165) BVM. Management of pancreaticoduodenal artery aneurysm associated with coeliac artery stenosis. Ann R Coll Surg Engl. 2019;101:e105-e107.
15. Kubota K, Shimizu A, Notake T, Wada Y, [Soejima](https://pubmed.ncbi.nlm.nih.gov/?sort=date&term=Soejima+Y&cauthor_id=36643356) Y. Treatment strategies for unruptured pancreaticoduodenal artery aneurysms associated with celiac artery occlusion. Ann Gastroenterol Surg. 2022;7:182-189.
16. Kwag M, Jung HS, Heo YJ, Baek JW, Shin GW. Embolization of Inferior Pancreaticoduodenal Artery Aneurysm with Celiac Stenosis or Occlusion: A Report of Three Cases and a Review of Literature. Taehan Yongsang Uihakhoe Chi. 2020;81:945-952.
17. Lim J, De Robles MS, [Putnis](https://pubmed.ncbi.nlm.nih.gov/?sort=date&term=Putnis+S&cauthor_id=36413038) S. Pancreaticoduodenal artery aneurysm associated with coeliac stenosis: a potential bomb that carries no warning. ANZ J Surg. 2023;93:1367-1368.
18. Miyahara K, Hoshina K, Nitta J, Kimura M, Yamamoto S, Ohshima M. Hemodynamic Simulation of Pancreaticoduodenal Artery Aneurysm Formation Using an Electronic Circuit Model and a Case Series Analysis. Ann Vasc Dis. 2019;12:176-181.
19. Sutton D, Lawton G. Coeliac stenosis or occlusion with aneurysm of the collateral supply. Clin Radiol. 1973;24:49-53.
20. Takeuchi Y, [Morikage](https://pubmed.ncbi.nlm.nih.gov/?sort=date&term=Morikage+N&cauthor_id=28238917) N, Samura M, Harada T, Yamashita O, Suehiro K, Okada M, Hamano K. Treatment Options for Celiac Stenosis and Pancreaticoduodenal Artery Aneurysms. Ann Vasc Surg. 2017:41:281.e21-281.e23.
21. Uher P, Nyman U, [Ivancev](https://pubmed.ncbi.nlm.nih.gov/?sort=date&term=Ivancev+K&cauthor_id=7580788) K, Lindh M. Aneurysms of the pancreaticoduodenal artery associated with occlusion of the celiac artery. Abdom Imaging. 1995;20:470-3.
22. Zhang XZ, Zhang W, Zhou W, Zhou W. Endovascular Treatment of Ruptured Pancreaticoduodenal Artery Aneurysm with Celiac Axis Stenosis. Ann Vasc Surg. 2019;57:273.e1-273.e5.
